# Supplementary material for: MetaRibo-Seq measures translation in microbiomes
Source: Nat Commun. 2020 Jun 29;11:3268. doi: 10.1038/s41467-020-17081-z (PMC7324362; doi:10.1038/s41467-020-17081-z)
Supplement: Supplementary file 10 — Supplementary Data 7 [file 41467_2020_17081_MOESM10_ESM.zip › File2/Confidence_VeryHigh_Taxonomy/82134_out.krona.html]

Javascript must be enabled to view this page.

members
magnitude
magnitudeUnassigned
count
unassigned
taxon
rank

82134\_out

4

2
4
superkingdom

phylum
1239
4

4
186801
class

order
186802
4

2
186806
family

2
1730
genus


SRS015431\_contig\_number\_88483SRS019068\_contig\_number\_105280
species
2
1262878

2
541000
family

2
1898205

SRS024663\_contig\_number\_contig-100\_4923.68271SRS893383\_contig\_number\_contig-100\_3058.153112
species
